# The complete chloroplast genome of an endangered and endemic species, *Acer yangbiense* (Aceraceae)

**DOI:** 10.1080/23802359.2019.1699878

**Published:** 2019-12-12

**Authors:** Li-Zhen Ling, Shu-Dong Zhang

**Affiliations:** School of Biological Sciences and Technology, Liupanshui Normal University, Liupanshui, China

**Keywords:** *Acer yangbiense*, Aceraceae, chloroplast genome, phylogenetic analysis

## Abstract

*Acer yangbiense* Y.S. Chen & Q.E. Yang is a species of Aceraceae with a very restricted distribution in Yunnan, China. In this study, the complete chloroplast (cp) genome sequence of *A. yangbiense* was reported and characterized. The cp genome is 155,706 bp in length and contains a pair of inverted repeats (IRs, 25,858 bp) separated by a large (85,859 bp) and small (18,131 bp) single-copy regions. A total of 128 genes were predicted, including 83 protein-coding genes, 37 tRNA genes and 8 rRNA genes. The phylogenetic analysis suggested that *A. yangbiense* is sister of the clade formed by *A. laevigatum*, *A. palmatum*, *A. wilsonii*, and *A. buergerianum*.

*Acer yangbiense* Y.S. Chen & Q.E. Yang belongs to the Aceraceae family and is a new maple species described in 2003 (Chen et al. 2003). It is rare and geographically restricted to Cangshan Mountains of Yunnan Province, China. This species is exposed to extinction because of its small population size, poor reproduction, and habitat degradation (Yang et al. 2015). *Acer yangbiense* is considered a critically endangered (CR) species (Gibbs and Chen 2009), and classified as a plant species with extremely small populations (PSESP) by the Chinese government and included in the PSESP rescue plan (Ma et al. 2013). To promote the conservation of this species, we sequenced and analyzed the complete chloroplast (cp) genome of *A. yangbiense* using high-throughput sequencing technology.

The fresh leaves of *A. yangbiense* were collected from Kunming Institute of Botany (N25°08′11″, E102°44′23″, 1,950 m), Yunnan, Southwest of China. The voucher specimen (lpssy0309) was deposited in the herbarium of the Liupanshui Normal University (LPSNU). Total genomic DNA was isolated and used for sequencing on the Illumina HiSeq 4000 Platform as previously described (Zhang et al. 2019). Approximately 2 Gb raw data were generated and used for *de novo* cp genome assembly with SPAdes (Bankevich et al. 2012) and all predicted genes were annotated using PGA (Qu et al. 2019). The complete cp genome sequence of *A. yangbiense* was deposited in GenBank database under accession number MN652924.

The complete cp genome of *A. yangbiense* is 155,706 bp in length, including a large single copy (LSC) region of 85,859 bp, a small single copy (SSC) region of 18,131 bp, and a pair of inverted repeats (IRs) of 25,858 bp each. The cp genome shows the GC content of 38.0% and contains 128 genes, including 83 protein-coding genes (PCGs), 37 transfer RNA (tRNA) genes, and 8 ribosomal RNA (rRNA) genes. Of them, 16 genes (*ndhB*, *rpl23*, *rps12*, *rps7*, *rrn16*, *rrn23*, *rrn4.5*, *rrn5*, *trnA*-*UGC*, *trnI*-*CAU*, *trnI*-*GAU*, *trnL*-*CAA*, *trnN*-*GUU, trnR-ACG*, *trnV*-*GAC* and *ycf2*) have two copies. Fifteen genes (*atpF*, *ndhA*, *ndhB*, *petB*, *petD*, *rpl16*, *rpl2*, *rpoC1*, *rps16*, *trnA-UGC*, *trnG-UCC*, *trnI-GAU*, *trnK-UUU*, *trnL-UAA*, and *trnV-UAC*) contain one intron and three genes (*clpP*, *rps12* and *ycf3*) have two introns.

The Aceraceae family comprises two genera (*Dipteronia* and *Acer*) with more than 200 species. To determine the phylogenetic position of *A. yangbiense*, the cp genomes of this species and previously released species of Aceraceae were used for phylogenetic reconstruction. In this study, the cp genomes from 11 representative species from the genus of *Acer* were downloaded and their GenBank accession numbers are provided in Figure 1. Two species from *Dipteronia* (*D. sinensis* and *D. yeriana*) were used as the outgroups. The complete cp genome sequences were aligned using MAFFT version 7.0 (Katoh and Standley 2013). Phylogenomic analysis was performed with the maximum likelihood (ML) and Bayesian inference (BI) methods (Ronquist and Huelsenbeck 2003; Stamatakis 2014). The phylogenetic analysis showed that *A. yangbiense*, *A. laevigatum*, *A. palmatum*, *A. wilsonii* and *A. buergerianum* form a monophyletic clade and *A. yangbiense* is sister of the other species of this clade (Figure 1).

**Figure 1.**
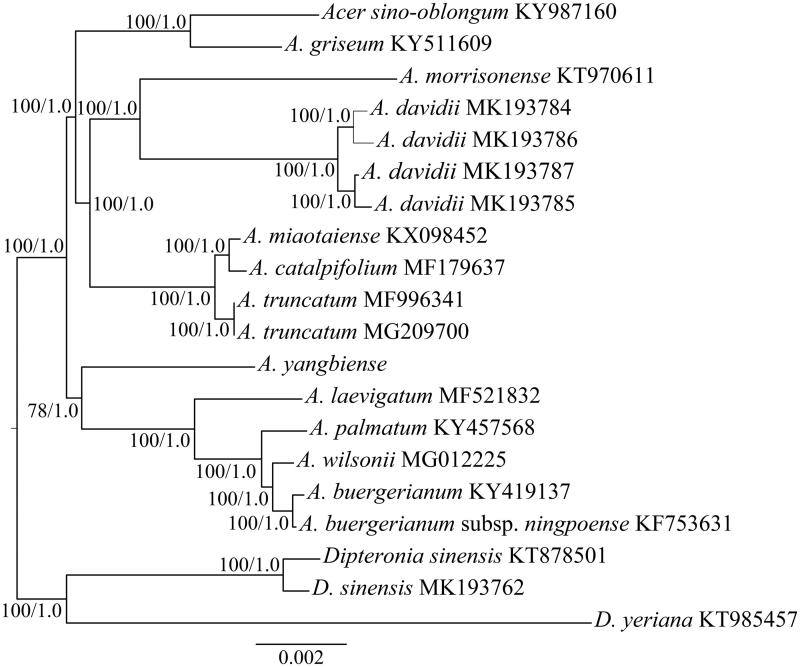
The maximum likelihood (ML) tree of *Acer* inferred from the complete chloroplast genome sequences. Numbers at nodes correspond to ML bootstrap percentages (1,000 replicates) and Bayesian inference (BI) posterior probabilities.
